# Navigating Solitary Rectal Ulcer Syndrome: A Surgeon’s Conundrum With Rectal Carcinoma in a Young Adult

**DOI:** 10.7759/cureus.68513

**Published:** 2024-09-03

**Authors:** Tushar Dahmiwal, Darshana Tote, Anup Zade, Abhilasha Bhargava, Yogesh B Manek, Srinivasa Reddy, Amol Gupta

**Affiliations:** 1 General Surgery, Jawaharlal Nehru Medical College, Datta Meghe Institute of Higher Education and Research, Wardha, IND

**Keywords:** bleeding per rectum, carcinoma rectum, constipation, rectal ulcer, solitary rectal ulcer

## Abstract

Solitary rectal ulcer syndrome is a rare, chronic, and benign disorder. It can be observed as ulcers in the rectal mucosa, solitary or multiple lesions. It can often be misdiagnosed with other intestinal morbidities, due to its clinical similarities. It can be diagnosed by clinical symptoms, radiological tools, and histopathological examination. Management is carried out by conservative methods such as lifestyle and dietary modifications with medical and surgical therapy. This is a case of a 37-year-old female with a major complaint of per-rectal bleeding. Proctoscopy revealed an irregular-ulcerated mass in the rectum, which was managed conservatively. The patient showed improved symptoms after a colonoscopy, at a six-month follow-up.

## Introduction

Solitary rectal ulcer syndrome (SRUS) is defined by a specific set of symptoms, endoscopic observations, and histological irregularities, and is considered an uncommon, non-malignant, and enduring ailment [1]. Although Cruveilhier initially described it in 1829, it was acknowledged in 1969 after Madigan and Morson assessed this illness's clinical and pathological aspects [2]. The name 'SRUS' is deceptive, suggesting that endoscopic intervention findings are limited to just one rectal ulcer. As the name implies, solitary ulcers are seen in only 20% of patients, whereas multiple ulcers are noted in 40% of patients. Lesions in the remaining patients range from broad-based polypoid lesions to single or numerous ulcers and mucosal erythema alone [3]. Estimates suggest that the yearly occurrence of SRUS is approximately 1 in 100,000 individuals, impacting both males and females, although it is less predominant among females [4]. Age at diagnosis varies from 14 to 76 years, with the median age being 48 years [3]. Clinical manifestations consist of rectal bleeding (56%), difficulty during bowel movements (28%), a sensation of pelvic fullness (23%), significant mucus discharge (23%), along with perineal and abdominal discomfort, prolonged excessive straining, a feeling of incomplete evacuation, constipation, and, on rare occasions, rectal prolapse [5]. Conversely, up to 26% of patients may not exhibit any symptoms [3,6-8]. This is a case report of a patient with SRUS that mimics rectal cancer in a young adult.

## Case presentation

A 37-year-old female patient visited the Surgical Outpatient Department complaining of abdominal pain and episodes of per-rectal bleeding that had become more intense during the previous two months. The bleeding was fresh, sparse, painless, and typically occurred after a bowel movement. Fever, self-digitation, or tenesmus were not linked to bleeding. She has been using oral laxatives, actively, for constipation for the last two years. She also lost 7 kg of weight inadvertently over the previous five months. There was no prior history of drug or food allergies, diabetes mellitus, hypertension, or any other comorbidities, and no surgical history. Family history of stomach cancer, carcinoma rectum, or similar complaints was negative. Vital signs on general assessment were within normal limits. Abdominal examination revealed a soft abdomen with mild epigastric pain and normal bowel sounds. A proctoscopy examination showed a large, irregular-ulcerated mass in the posterior wall of the rectum. A blood test for faecal occult was negative. The laboratory findings are mentioned in Table 1.

**Table 1:** Laboratory findings of the patient g/dL: Grams/decilitre; µL: Microlitre; mg/dL: Milligrams/decilitre; mmol/L: Millimoles/litre; ng/ml: Nanograms/millilitre; IU/mL: International unit/millilitre; AU/mL: Arbitrary units/millilitre; pg/mL: Picograms/millilitre

Parameter	Patient’s value	Normal range
Haemoglobin	7.8 g/dL	13-17 g/dL
White blood cell count	9.1 thousand/µL	4.0-11.0 thousand/µL
Serum creatinine	1.1 mg/dL	0.66-1.25 mg/dL
Serum sodium	138 mmol/L	135-145 mmol/L
Serum potassium	3.5 mmol/L	3.5-5.2 mmol/L
Carcinoembryonic antigen (CEA)	2.6 ng/mL	0-5.0 ng/mL
Carbohydrate antigen 19.9 (CA19.9)	0.9 IU/mL	<37.0 IU/mL
Antinuclear antibodies (ANAs)	8.93 AU/mL	32 AU/mL
Vitamin B12	900 pg/mL	150-980 pg/mL

**Figure 1:**
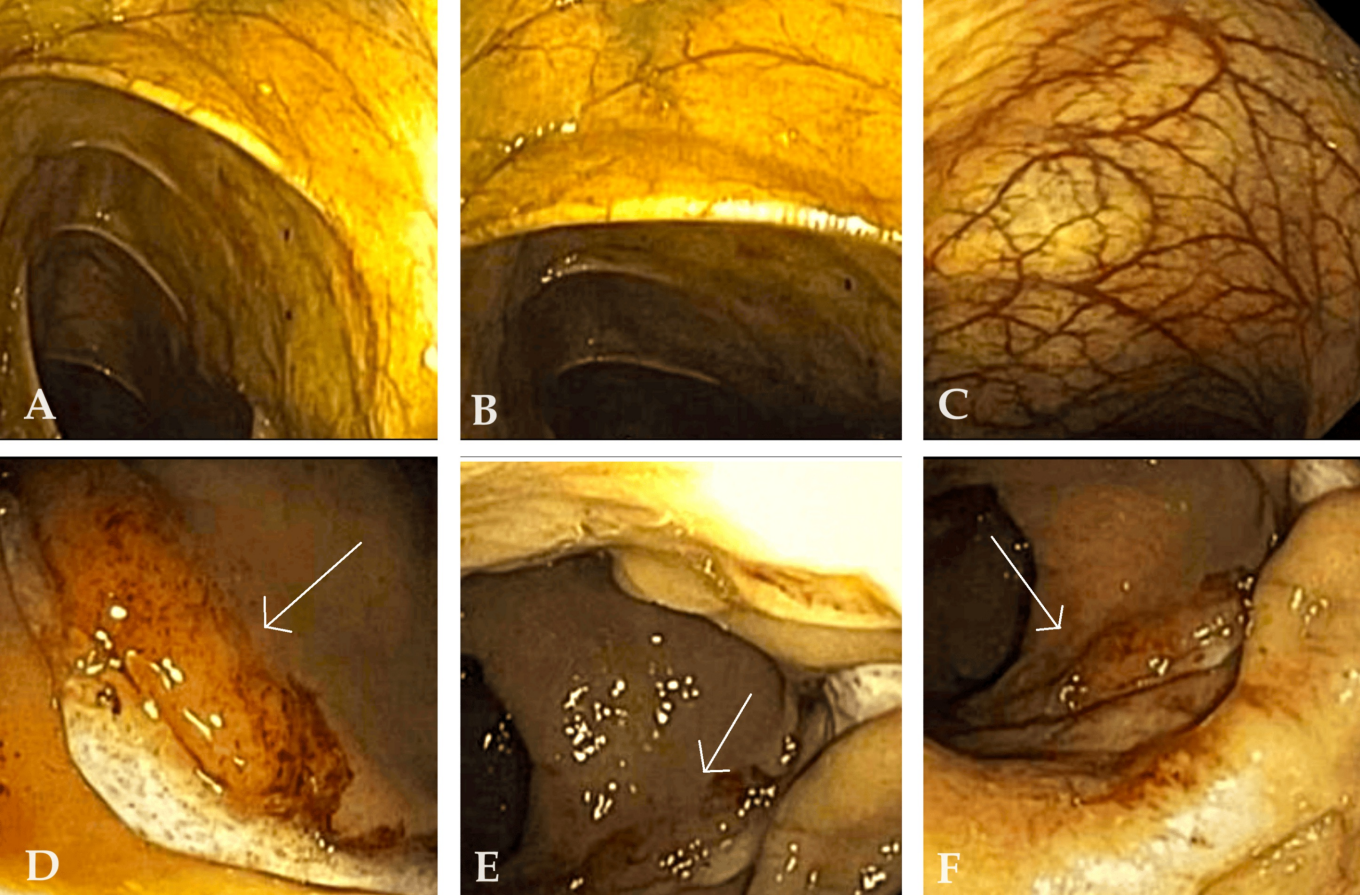
Colonoscopy findings (A)-(C): Areas of the colon which were normal; (D): Linear ulcerations; (E)-(F) Linear ulcerations covered with slough in the lower part of the anal canal

The patient was subjected to a colonoscopy, which showed two linear ulcerations covered with slough in the lower part, located around 4 cm above the anal rim. The ulcerated mass underwent a biopsy and was sent for histopathological examination (Figure 1).

**Figure 2:**
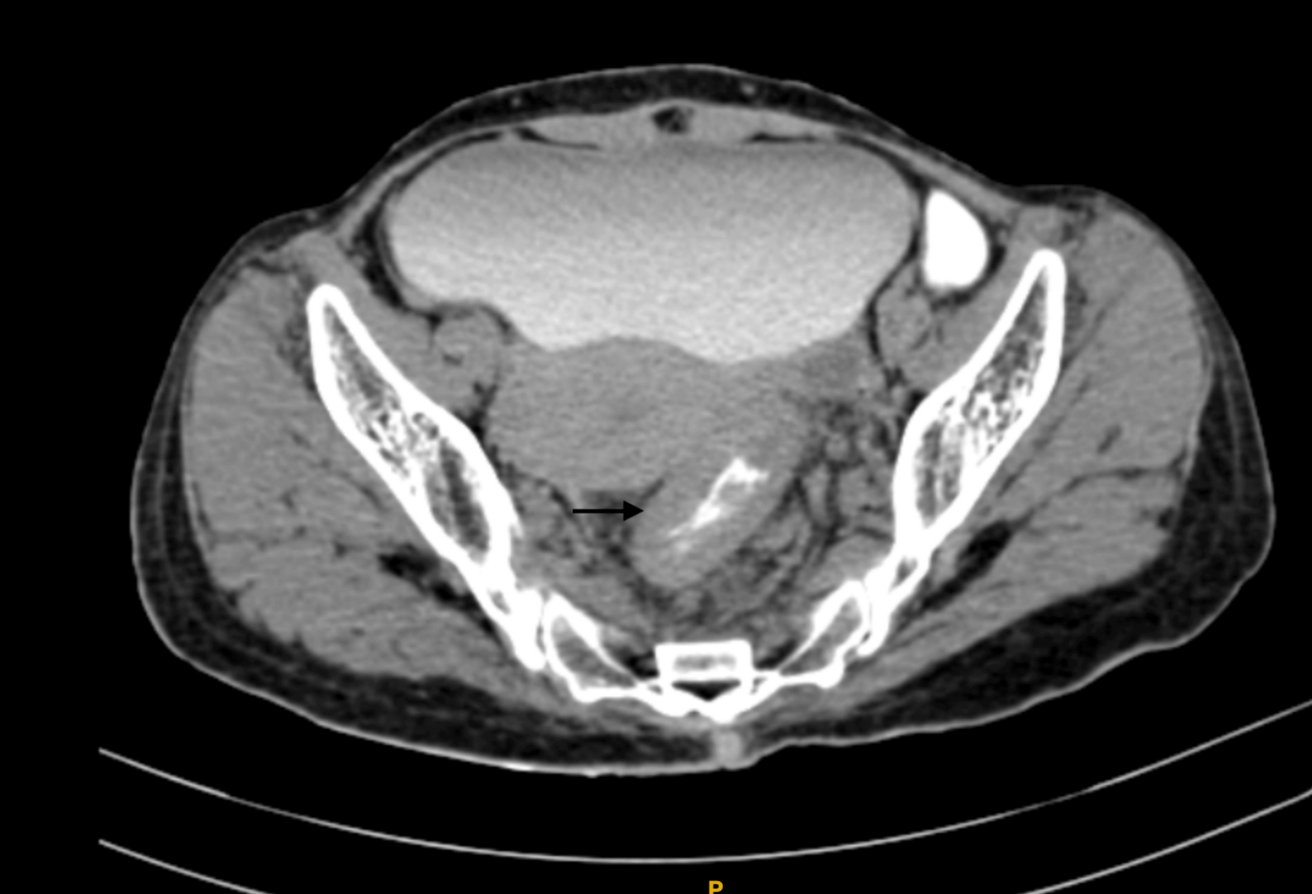
Abdominopelvic CECT scan findings The black arrow shows irregular circumferential wall thickening in the rectum, involving the rectosigmoid junction causing luminal narrowing. CECT: Contrast-enhanced computed tomography

The rest of the cecum, and the colon along the sigmoid colon, showed no abnormality. An abdominopelvic contrast-enhanced computed tomography (CECT) scan showed a heterogeneously enhanced irregular circumferential wall thickening in the mid and lower rectum. It involved the recto-sigmoid junction, with adjacent peri-rectal fat stranding, sub-centimetric lymph nodes, and peri-rectal collaterals (Figure 2).

**Figure 3:**
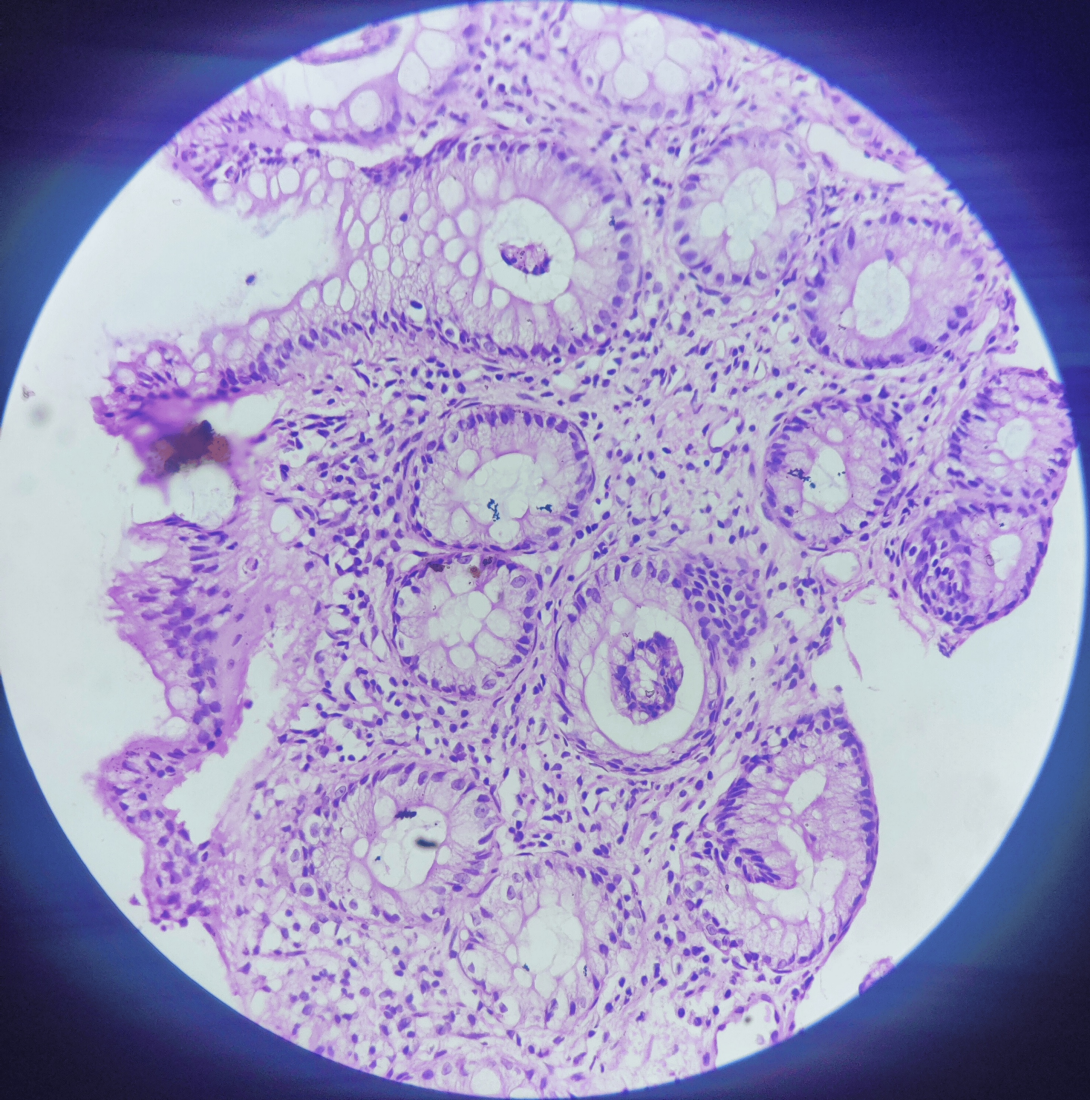
Haematoxylin and eosin-stained histopathological slide showing smooth muscle proliferation and colonic crypts surrounded by fibroblasts at 20x magnification

The biopsy findings revealed colonic fragments showing surface erosions. The underlying crypts appeared remarkable. Lamina propria showed ectatic capillaries, fibromuscular obliteration, and focal splaying of muscle fibres from muscularis mucosae. Mild infiltration of lymphocytes, plasma cells, and eosinophils was observed within lamina propria. The histopathological section from the rectal biopsy showed haphazardly arranged benign colonic crypts surrounded by fibroblasts, and smooth muscle proliferation (Figure 3).

No dysplasia or invasions were identified, which was suggestive of SRUS. The patient opted for conservative management for four months with laxatives and analgesics, along with dietary modifications, and clinical symptoms improved marginally. A follow-up colonoscopy showed significant lesion resolution, with minimal signs of ulcerative scarring remaining.

## Discussion

The average duration interval between the onset of symptoms and a proper diagnosis in SRUS cases is five years, with adult cases falling between 3 months and 30 years. However, the time interval for paediatric patients is comparatively less (1.2-5.5 years) [9]. In addition to tenesmus, perineal and abdominal pain, abundant mucous discharge, fresh (bright) rectal bleeding, and occasionally rectal prolapse are typically reported. The indications and manifestations, such as self-digitation to eliminate impacted faeces or reduce rectal prolapse, align with a background of prolonged intense straining, constipation, or irregular bowel movements. While the exact cause and pathophysiology of SRUS remain unclear, several theories have been put forth, including excessive effort during bowel movements, telescoping of the rectal lining, irritation caused by firm stool or manual manoeuvres, and persistent injury to the rectal lining due to prolonged straining [10]. Paradoxical contraction of the puborectalis muscle while defecating is also a factor in the pathogenic process of obvious or hidden rectal prolapse over time, leading to ischemic changes and ulcers in the rectal mucosa. This is supported by observations of anorectal physiology, showing dyssynergia present in 25%-82% of SRUS patients [11]. Another significant aspect involved in SRUS incidence is excessive strain during defecation and mucosal irritation as a result of hard-impacted faeces.

Diagnostic methods involve clinical observations, proctosigmoidoscopy, histological analysis, magnetic resonance imaging (MRI), and anorectal functional studies, such as manometry [12]. The name ‘SRUS’ can be misleading, as it is not always present in solitary form; there can be multiple lesions involved, which might lead a clinician to consider more common diagnoses, such as ischemic colitis, rectal carcinoma, and inflammatory bowel disease (IBD) (including Crohn’s disease and ulcerative colitis), that align better with the clinical symptoms. A research study of 80 SRUS patients revealed a range of macroscopic lesions, including polypoid lesions in 44% (most prevalent in asymptomatic cases), ulcerated lesions in 29%, and oedematous, hyperaemic, non-ulcerated mucosa in 27% of the cases [3]. There are factors, such as inadequate rectal biopsy or unrecognized associated histological characteristics, leading to delayed or inaccurate diagnosis. Other minor microscopic abnormalities, like surface erosions, moderate inflammation, deformed crypts, and reactive epithelial atypia, can lead to misdiagnosis of IBD and cancer.

Nevertheless, abnormal extensions of smooth muscle fibres and extensive collagen accumulation in the lamina propria would serve as critical histological indicators [13,14]. The ulcer features noted in this case were similar to other documented reports in size and location. The ulcer measured 1 x 1 cm and was situated 5 cm above the anal verge on the posterior rectal wall, with research literature mentioning the size of the ulcers in a range between 0.5 and 4 cm in diameter and located 3-10 cm above the anal margin [2,6]. Fibromuscular obliteration of the lamina propria, hypertrophied muscularis mucosa with muscle fibres extending upwards (between the crypts), and abnormal glandular crypts are some of the key histological alterations linked to SRUS, which were noted in this case as well [15]. Imaging tests (MRI/CT) initially suggested the presence of malignancy in this case, but the biopsy results ultimately proved otherwise, leading to an early diagnosis of SRUS. The primary treatment approaches consist of four key pillars: pharmacological therapy, biofeedback therapy, surgical intervention, and conservative management, encompassing modifications in nutrition, behaviour, and hygiene practices. People with only mild to moderate symptoms and no significant mucosal prolapse typically show positive responses to changes in diet and behaviour, which was also noted in this case, as evidenced by follow-up colonoscopy findings. This therapeutic approach may not provide advantages for individuals experiencing moderate to severe rectal intussusception, considerable fibrosis and inflammation, and/or reducible external prolapse.

Furthermore, conservative treatment may include administering topical fibrin sealant, salicylates, corticosteroids, sulfasalazine, mesalazine, and sucralfate for symptomatic alleviation [14]. Biofeedback looks promising in cases that are non-responsive to conservative therapy. It corrects aberrant pelvic floor behaviour, which in turn helps reduce excessive stress during defecation, with improvement noted in patients with dyssynergia defecation [16]. Cases with no improvement noted by conservative management or rectal prolapse may consider surgical intervention. During surgical procedures, ulcer excision and mucosal resection (Délorme’s operation) or peritoneal proctectomy (Altemeier’s method) are used [13,17]. In the absence of rectal prolapse in this case, conservative treatment, including dietary modifications, lifestyle changes, and the administration of stool-softening medications, was primarily considered. It is crucial to emphasize the importance of early diagnosis, as evidenced by the improvement in her clinical condition at four- and six-month follow-ups. This can help protect the individual from unnecessary medical and surgical procedures that are inherently risky.

## Conclusions

Conservative management of SRUS, including dietary and lifestyle adjustments, is primarily recommended. A heightened level of suspicion is essential to facilitate the early histopathological diagnosis of this condition, due to its highly similar clinical presentations to ischemic colitis, rectal cancer, and irritable bowel disease. Both pathologists and surgeons must maintain a high level of suspicion regarding SRUS as a potential differential diagnosis.

## References

[1] Felt-Bersma RJ, Tiersma ES, Cuesta MA (2008). Rectal prolapse, rectal intussusception, rectocele, solitary rectal ulcer syndrome, and enterocele. Gastroenterol Clin North Am.

[2] Madigan MR, Morson BC (1969). Solitary ulcer of the rectum. Gut.

[3] Tjandra JJ, Fazio VW, Church JM, Lavery IC, Oakley JR, Milsom JW (1992). Clinical conundrum of solitary rectal ulcer. Dis Colon Rectum.

[4] Martin CJ, Parks TG, Biggart JD (1981). Solitary rectal ulcer syndrome in Northern Ireland. Br J Surg.

[5] Suresh N, Ganesh R, Sathiyasekaran M (2010). Solitary rectal ulcer syndrome: a case series. Indian Pediatr.

[6] Tjandra JJ, Fazio VW, Petras RE, Lavery IC, Oakley JR, Milsom JW, Church JM (1993). Clinical and pathologic factors associated with delayed diagnosis in solitary rectal ulcer syndrome. Dis Colon Rectum.

[7] Bulut T, Canbay E, Yamaner S, Gulluoglu M, Bugra D (2011). Solitary rectal ulcer syndrome: exploring possible management options. Int Surg.

[8] Ong J, Lim KH, Lim JF, Eu KW (2011). Solitary caecal ulcer syndrome: our experience with this benign condition. Colorectal Dis.

[9] Dehghani SM, Malekpour A, Haghighat M (2012). Solitary rectal ulcer syndrome in children: a literature review. World J Gastroenterol.

[10] Dehghani SM, Haghighat M, Imanieh MH, Geramizadeh B (2008). Solitary rectal ulcer syndrome in children: a prospective study of cases from southern Iran. Eur J Gastroenterol Hepatol.

[11] Vaizey CJ, van den Bogaerde JB, Emmanuel AV, Talbot IC, Nicholls RJ, Kamm MA (1998). Solitary rectal ulcer syndrome. Br J Surg.

[12] Keshtgar AS (2008). Solitary rectal ulcer syndrome in children. Eur J Gastroenterol Hepatol.

[13] Edden Y, Shih SS, Wexner SD (2009). Solitary rectal ulcer syndrome and stercoral ulcers. Gastroenterol Clin North Am.

[14] Levine DS, Surawicz CM, Ajer TN, Dean PJ, Rubin CE (1988). Diffuse excess mucosal collagen in rectal biopsies facilitates differential diagnosis of solitary rectal ulcer syndrome from other inflammatory bowel diseases. Dig Dis Sci.

[15] Chiang JM, Changchien CR, Chen JR (2006). Solitary rectal ulcer syndrome: an endoscopic and histological presentation and literature review. Int J Colorectal Dis.

[16] Vaizey CJ, Roy AJ, Kamm MA (1997). Prospective evaluation of the treatment of solitary rectal ulcer syndrome with biofeedback. Gut.

[17] Beck DE (2002). Surgical therapy for colitis cystica profunda and solitary rectal ulcer syndrome. Curr Treat Options Gastroenterol.

